# Postoperative Dysphagia Subsequent to Posterior Fixation of an Axis Fracture Without Cranial Fixation: A Case Report

**DOI:** 10.7759/cureus.36664

**Published:** 2023-03-25

**Authors:** Takaki Yoshiyama

**Affiliations:** 1 Orthopedics, Osaka Red Cross Hospital, Osaka, JPN

**Keywords:** posterior cervical fixation, axis fracture, hangman’s fracture, dysphagia, surgical case report

## Abstract

The incidence of dysphagia following cervical fusion, which involves the occipital bone, is well established. However, dysphagia occurring after cervical fusion not involving the occipital bone is exceedingly rare. We present a case report of a 54-year-old male who developed unexplained dysphagia subsequent to posterior fusion up to C1-3 for an axis fracture.

## Introduction

It is widely recognized that postoperative dysphagia should be taken into consideration during cervical fusion, especially when the occipital bone is involved. These occurrences have been studied in some depth, with particular emphasis on maintaining a postoperative occipito-C2 (O-C2) angle that exceeds the preoperative angle [1-3]. Similarly, the use of the swallow-line (S-line) criteria is a well-established standard for postoperative swallowing dysfunction [4]. Nevertheless, there are limited reports of dysphagia following posterior cervical fusion that does not involve the occipital bone.

## Case presentation

**Figure 1:**
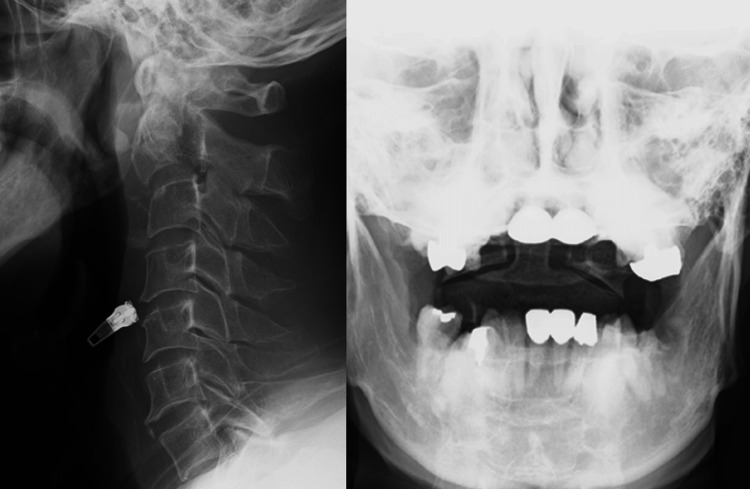
Preoperative plain X-ray images Displacement of C2 with angulation is observed. This fracture type is unstable.

**Figure 2:**
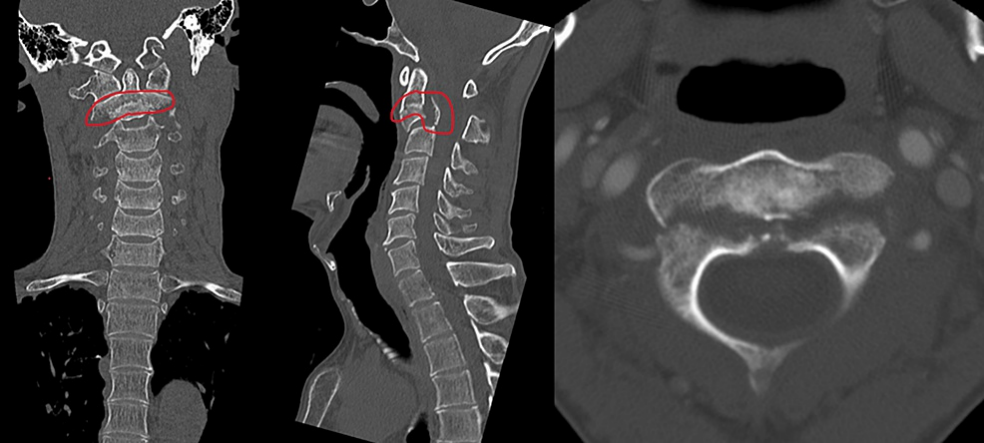
Plain CT images Plain CT showed fractures in the vertebral bodies as well as the intervertebral joints. Therefore, we determined that this patient required fixation, including C1.

**Figure 3:**
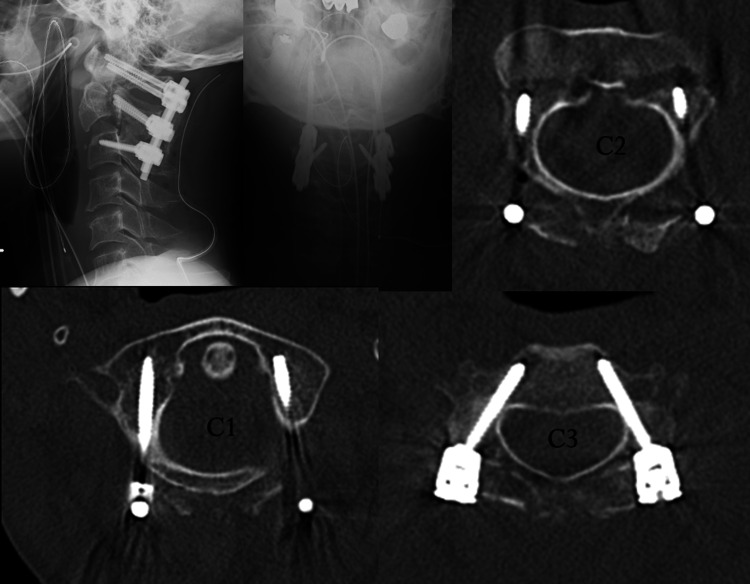
Postoperative plain X-ray and CT images No implant deviation or obvious protrusion into the vertebral artery.

The patient, a current smoker aged 54 years, has a 36-pack-year smoking history. One week prior to the presentation, he sustained a fall down a flight of stairs at his residence. As his neck pain persisted, he sought medical attention and was subsequently diagnosed with an axis vertebra fracture by his primary care provider, who promptly referred him to our hospital for further management. On evaluation, he demonstrated a limited range of motion attributed to neck pain. However, there were no discernable neurological deficits, including motor weakness, sensory disturbances, abnormal reflexes, or dysphagia. Given the presence of fracture lines involving both the pedicle of the axis vertebra and the vertebral body (Hangman fracture, Levine-Edwards classification type II + Anderson classification of odontoid fractures type III), a decision was made to proceed with posterior fixation up to C1-3 (Figures 1-3).

The operation was conducted one week after the patient's admission to the hospital. The procedure was completed without any complications, and the patient was transferred to the general ward. However, on the first postoperative day during dinner, the patient displayed clear signs of aspiration and reported difficulty in swallowing. Later that same night, the patient experienced difficulty in expectorating sputum, a decrease in oxygen saturation (SpO_2_) levels, and plain computed tomography (CT) scan revealed obvious pneumonia. We concluded that the patient was suffering from dysphagia and respiratory failure due to aspiration pneumonia, and as a result, he was reintubated and moved to the intensive care unit (ICU). The patient had a significant amount of sputum, which may have been due to his smoking history, and required a tracheostomy seven days after intubation. He was placed on continuous positive airway pressure (CPAP) and transferred back to the general ward on the 14th postoperative day. The patient was weaned off the ventilator entirely by the 20th postoperative day. Direct swallow training commenced on the 32nd postoperative day, and with the assistance of a speech therapist, the patient's diet was gradually upgraded to solid food. The patient was able to consume regular food by the 59th postoperative day and was eventually discharged from the hospital on the 70th postoperative day.

## Discussion

The patient suffered from dysphagia following C1-3 fusion surgery, which fortunately improved with conservative treatment. None of our medical team anticipated the occurrence of postoperative dysphagia and thus investigated the possible cause. Reports have indicated instances of hypoglossal neuropathy following cervical spine surgery, including posterior cervical spine surgery, known as Tapia syndrome [5-7]. This syndrome may result from blood flow disturbance due to intubation tubes in the ascending pharyngeal artery, which supplies the hypoglossal and vagus nerves. However, unlike these cases, the present case did not exhibit tongue deviation when parroting. Another possibility was the occlusion of the vertebral artery during surgery, resulting in a brainstem infarction or lateral medullary syndrome, commonly known as Wallenberg syndrome. However, brainstem magnetic resonance imaging and magnetic resonance angiography showed no abnormalities or obvious causes. Additionally, there was no physical stenosis of the larynx or pharynx.

An otolaryngologist was consulted for the patient, but despite observing decreased laryngeal perception and dysphagia during the pharyngeal phase of swallowing, the cause could not be determined. It is postulated that the traction maneuver performed during surgery may have temporarily reduced blood flow to the vertebral artery, leading to the possibility of transient brainstem ischemia. However, radiological imaging studies were inconclusive in determining the cause. In cases where the aforementioned causes are not recognized, conservative treatment has been reported to lead to the recovery of swallowing function, as seen in a past case report [8]. The occurrence of dysphagia of unknown origin after cervical fusion surgery (C1-3 fusion) that does not involve the occipital bone is rare, and therefore, the treatment course for this case is deemed noteworthy to report.

## Conclusions

Dysphagia may manifest in patients even after cervical fusion that does not entail the occipital bone. Thus, screening for hypoglossal nerve palsy or brainstem stroke is recommended. In the absence of any discernable underlying pathology, conservative treatment is indicated, with emphasis on swallowing rehabilitation to achieve functional recovery.
